# An Overview of Chronic Disease Models: A Systematic Literature Review

**DOI:** 10.5539/gjhs.v7n2p210

**Published:** 2014-10-28

**Authors:** Ashoo Grover, Ashish Joshi

**Affiliations:** 1 Indian Council of Medical Research, Government of India, New Delhi, India; 2 Center for Global Health and Development, College of Public Health, University of Nebraska Medical Center, Omaha, Nebraska, USA

**Keywords:** Chronic Disease Model, elements, CVD, Diabetes and COPD

## Abstract

**Aims:**

The objective of our study was to examine various existing chronic disease models, their elements and their role in the management of Diabetes, Chronic Obstructive Pulmonary Disease (COPD), and Cardiovascular diseases (CVD).

**Methods:**

A literature search was performed using PubMed and CINHAL during a period of January 2003- March 2011. Following key terms were used either in single or in combination such as “Chronic Disease Model” AND “Diabetes Mellitus” OR “COPD” OR ‘CVD”.

**Results:**

A total of 23 studies were included in the final analysis. Majority of the studies were US-based. Five chronic disease models included Chronic Care Model (CCM), Improving Chronic Illness Care (ICIC), and Innovative Care for Chronic Conditions (ICCC), Stanford Model (SM) and Community based Transition Model (CBTM). CCM was the most studied model. Elements studied included delivery system design and self-management support (87%), clinical information system and decision support (57%) and health system organization (52%). Elements including center care on the patient and family (13%), patient safety (4%), community policies (4%), built integrated health care (4%) and remote patient monitoring (4%) have not been well studied. Other elements including support paradigm shift, manage political environment, align sectoral policies for health, use healthcare personnel more effectively, support patients in their communities, emphasize prevention, identify patient specific concerns related to the transition process, and health literacy between visits and treatments have also not been well studied in the existing literature.

**Conclusions:**

It was unclear to what extent the results generated is applicable to different populations and locations and therefore is an area of future research. Future studies are also needed to test chronic disease models in settings where more racially and ethnically representative patients receive chronic care. Future program development should also include information on other barriers including transportation issues, finances and lack of services.

## 1. Introduction

Chronic diseases are diseases of long duration and generally slow progression. As per World Health Organization (WHO), the four main types of chronic diseases are cardiovascular diseases (like heart attacks and stroke), cancer, chronic respiratory diseases (such as chronic obstructed pulmonary disease and asthma) and diabetes (Alwan, 2011). Chronic diseases are by far the leading cause of death in the world, representing over 60% of all annual deaths. Of the 57 million deaths that occurred globally in 2008, 36 million were due to chronic diseases comprising mainly cardiovascular diseases, diabetes, chronic lung diseases and cancers (Alwan et al., 2010) About one fourth of global chronic disease related deaths took place before the age of 60 years. Some 80% of all chronic disease deaths occurred in low- and middle-income countries. The burden of chronic diseases is rising fastest among lower-income countries, populations and communities and is projected to increase substantially over the next 2 decades (Ezzati, Lopez, Rodgers, & Murray, 2005).

Diabetes represents a significant public health problem worldwide by decreasing quality of life and causing death and disability at great economic cost. Though quality diabetes care is essential to prevent long term complications, care often falls below recommended standards regardless of health care setting or patient population, emphasizing the necessity for system change. Cardiovascular disease (CVD) is the leading cause of death worldwide accounting for approximately 18 million deaths a year (Ezzati et al., 2005). CVD is also the leading cause of mortality in developing countries. Mortality from ischemic heart disease in developing countries is expected to increase by 120% for women and 137% for men (Leeder, Raymond, Greenberg, Liu & Esson, 2004). The respiratory diseases, including asthma and chronic obstructive pulmonary disease (COPD), caused 4.2 million deaths in 2008 and 90% deaths occurred in low and middle income countries (Ezzati et al., 2005).

The World Health Organization estimates that there will be a significant economic impact of chronic diseases worldwide. In 2005, the estimated loss in national income from heart disease, stroke and diabetes was 18 billion dollars in China, 11 billion dollars in Russian Federation, 9 billion dollars in India, and 2.7 billion dollars in Brazil. Similarly, the losses for UK, Pakistan, Canada, Nigeria and the United Republic of Tanzania were 1.6 billion dollars, 1.2 billion dollars, 0.53 billion dollars, 0.4 billion dollars and 0.1 billion dollars respectively. Three quarters of health care expenditure in United States is on chronic disease bills (US $ 1-7 trillion per year) (WHO, 2011). The results indicate that the burden of chronic diseases poses appreciably greater constraints to economic performance in low and middle income countries. The estimates do not include the life-time cost of morbidity, disabilities, and foregone expected lifetime earnings of individuals (Abegunde & Stanciole, 2006).

Age related changes, complicated by multiple, progressive physical, cognitive and emotional health problems contribute to accelerate functional decline, poorer quality of life and decreased survival rates. The constraints on limited resources, time and adequate information further adds to the challenge for the decision making process. Decision-makers at the population and individual levels each need to choose the best intervention for a specific health problem and this is particularly true for chronic diseases (Wagner, Davis, Schaefer, Von, & Austin, 2002). Chronic disease management has been a difficult challenge because of several factors including lack of information technology in outpatient settings; multiple sources of nonintegrated information; limited access to and use of specialists including education services; and time constraints.

Addressing increased incidence of chronic disease is one of the most important challenges for the health system. In contrast to the traditional medical model management of acute conditions, management of chronic disease requires that patients take a more active role in the day-to-day decisions about the management of their illness. This new disease paradigm requires that there be a working patient-provider partnership that involves effective treatment within an integrated system of collaborative care. The essential ingredient of effective chronic care management is the partnership between the patient and health professionals because it offers the opportunity to empower patients to become more active in managing their health. When patients are more informed, involved, and empowered, they interact more effectively with healthcare providers and strive to take actions that will promote healthier outcomes (Bodenheime, Lorig, Holman, & Grumbach, 2002). The patient is central to defining the disease-related problems and the self-management program assists them with problem solving and gaining the self-efficacy and confidence to deal with the problems.

A large diversity of chronic disease models exist in the literature. Different models have different elements to consider. Some consider self-management; others have health systems approach, and few have community participation approach while others include selected chronic diseases treatments. However, model construction and development is complex and difficult. Critiquing and providing a comprehensive overview of all models is a challenging task.

The objective of our study was to systematically review and evaluate the strengths and limitations of existing chronic disease models and their applications towards the management of most common chronic diseases such as Diabetes, COPD and CVD.

## 2. Materials and Methods

### 2.1 Search Methodology

All searches in Pubmed and CINHAL were conducted with the following limits: date range from 1/1/2003 to 31/3/2011, search term used was “Chronic disease model” OR “Chronic Care Model” AND “Diabetes” OR “COPD” OR “Cardiovascular Diseases”; studies should focus on humans, be in English and should be either clinical trial, controlled clinical trial, randomized controlled trial, journal article, practice guideline, or government publications. A series of searches was conducted on MeSH entry terms. The inclusion criteria included articles that described the origin of the chronic disease model, its rationale and their elements.

**Figure 1:**
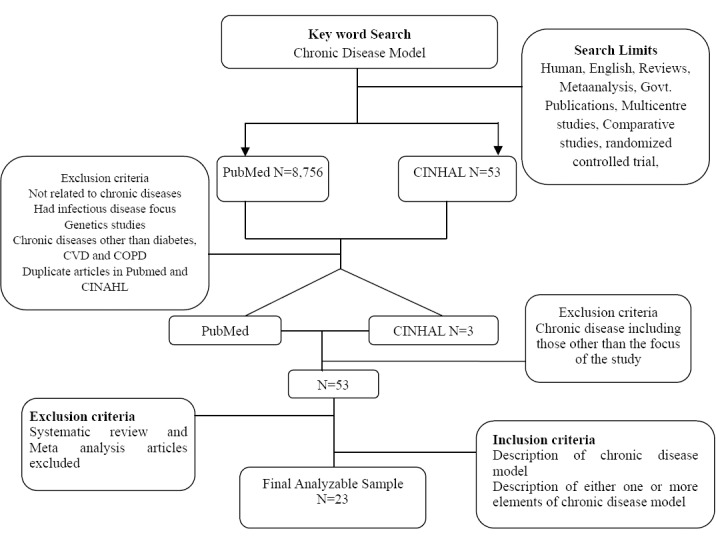
Overview of the search methodology

Exclusion criteria for the search terms included duplicate terms, not related to chronic diseases, had infectious disease focus, genetics studies and did not specify chronic disease model. Articles involving genetics, treatments, or biomarkers of chronic diseases were excluded as were case reports, meta-analyses, and reviews. The lists of articles retrieved were saved as text files and as saved searches within PubMed’s My NCBI feature. An overview of the search strategy is shown in Figure 1. Article lists were compiled using PubMed’s “Collections” feature in order to group all the articles and to eliminate duplicate articles.

### 2.2 Data Extraction

Table 1 shows variable extracted for the study.

**Table 1:** Information about the various variables extracted for the overview of chronic disease models

Study year	Information was recorded on the number of studies that were published during the various years from 2003-2011
Study location	Information was recorded on the location of the studies including U.S. versus non U.S. based and whether or not the studies were done in rural or urban settings.
Study design	Information was also recorded if the studies were observational or randomized controlled clinical trials and if they were interventional or not.
Studies follow up	The duration of the studies was also recorded to examine the impact of the chronic models on longitudinal
Disease studied	The review is focused on diseases including Diabetes, Chronic Obstructive Pulmonary Disease and Cardiovascular diseases because of their predominance in resulting death and disability worldwide.
Chronic disease model and its elements	Information was recorded on the specific chronic disease models and their elements that were described and evaluated across all these studies
Outcomes assessed	Information was also recorded about the various outcomes that were measured in these studies.

### 2.3 Statistical Analysis

Descriptive analysis was performed to report frequency analysis on the various variables that were extracted with a particular emphasis on the various chronic disease models and its elements. Additional analysis was performed to examine the distribution of the process, clinical and non-clinical variables. Stratified analysis was performed to determine frequency of the health outcomes studied. Stratified analysis was performed to examine the change in the various outcomes that were assessed. The stratification analysis was performed by chronic disease elements studied and the chronic diseases studied such as CVD, DM and COPD. All analysis was performed using SAS V9.1.

## 3. Results

The study identified 8,756 articles from PubMed search and CINHAL search resulted in 53 articles. After applying the relevant inclusion and exclusion criteria as described, 53 articles were found relevant to the study. The articles were reviewed and those which had included information about the specific chronic disease model and their associated elements were included in the final analysis resulting in an overall analyzable sample of 23 articles (Table 2).

**Table 2:** Summary of different elements of chronic disease models studied during 2003-2011 (Diabetes Mellitus, CVD and COPD). The X sign indicates that an element of a specific chronic disease model was studied primarily while + sign shows the presence of similar element being a part of other chronic disease model. The + sign shows an overlap of different elements for various chronic disease models

Chronic Disease Models	1	2	3	4	5	6	7	8	9	10	11	12	13	14	15	16	17	18	19	20	21	22	23	N
**Wagner CCM**																								
Health system or Health organization				+			X					X			X	X	X	X	X	X	X	X	X	11
Clinical Information System (CIS)	X	X		+		X						X			X	X	X	X	X		X	X	X	12
Decision support				+	X	X	X	X				X			X	X	X	X	X		X	X	X	13
Delivery system design	X		X	+	X	X	X	X	X	+		X	X	X	X	X	X	X	X	X	X	X	X	19
Self management support	X	X	X	+	X	X	X	X	X	+		X	X	X	X	X	X	X	X		X	X	X	19
Community linkages				+		X						X				X	X	X	X			X	X	8
**Improving Chronic Illness Care**																								
Patient safety (in Health System)				X																				1
Cultural competency (in Delivery System Design)	+			X																+				1
Care coordination (in Health System and Clinical Information Systems)				X	+									+						+				1
Community policies (in Community Resources and Policies)				X																				1
Case management (in Delivery System Design)				X										+										1
**Innovative Care for chronic conditions**																								
Support a paradigm shift																								0
Manage the political environment																								0
Build integrated health care																					X			1
Align sectoral policies for health																								0
Use healthcare personnel more effectively																								0
Center care on the patient and family									X			X		X										3
Support patients in their communities																								0
Emphasize prevention																								0
**Stanford Model**																								
Self Management	+	+	+		+	+	+	+		X		+	+	+	+	+	+	+	+		+			1
**Transition Care Model**																								
patient-specific concerns related to the transition process																								0
Medication adherence and persistence																								0
Health literacy between MD visits/treatment																								0
Remote patient monitoring											X													1

**Figure 2:**
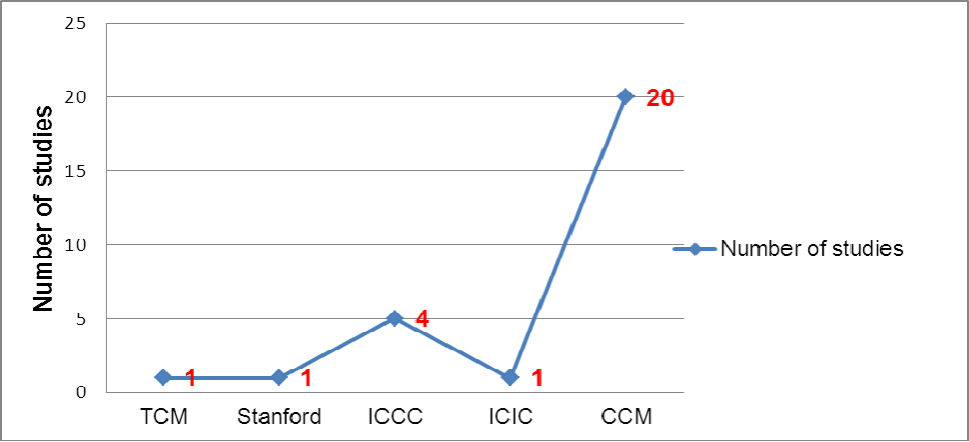
Distribution of number of studies based on type of chronic disease models used (Number more than 23 due to presence of 1 or more model in one study)

The majority of the articles were US-based (n=18/23), followed by New Zealand (n=2/23) and one each in Australia, Switzerland and Italy. More than half the studies were interventional (n=12/23), followed by cross sectional (n=10/23) and one study was a descriptive study (N=1). The average follow up period in these studies ranged from 18 weeks to 4 years. Only two studies were done in rural settings that implemented chronic disease models. The five chronic disease models managing Diabetes, COPD and CVD included chronic care model (CCM), Improving Chronic Illness Care (ICIC), Innovative Care for Chronic Conditions (ICCC), Stanford model and Transitional Care Model (TCM). CCM was the most studied chronic disease model (Figure 2). Each of them has been described in detailed as below.

### 3.1 Overview of Chronic Disease Models

(a) Chronic Care Model, CCM (Wagner, Austin & Von, 1996)

The CCM is a model of care designing essential elements of chronic disease care. The model provides guidance for a shift from an acute, episodic health system focus to one that is required for effective chronic disease care (Wagner et al. 2002; Bodenheime et al., 2002; Wagner et al., 1996; Wagner et al., 2001; Wagner, Austin, & Von, 1996). The model argues that real improvement in outcomes will occur only when clinical systems reconfigure themselves specifically to address the needs and concerns of chronically ill patients. The CCM applies to a broad range of chronic illnesses and serves as a roadmap for physicians to organize their practices and to meet the complex needs of chronically ill people. It provides a proactive; patient-centered, evidence-based approach. Within this model are the six major elements that interact to promote high quality care for patients with chronic disease. The 6 elements are described in Table 3.

**Table 3:** Description of Six elements of *CCM*

Element	Description
*Health system or a health organization*	Entity desiring to implement CCM is composed of staff, leaders, operations, values and goals of the organization and may vary from a small family practice to a multisite integrated health system.
*Clinical information systems (CIS*	Needs to have readily accessible disease specific database of individual patients and this database should alert the provider to needed tests and provide tracking. The system should facilitate and promote exchange of information between providers and patients.
*Decision support*	Defined as evidence based guidelines consistent with scientific evidence and patient preference. These guidelines should be embedded into daily practice and should be shared with patients to encourage participation.
*Delivery system design*	Involves how care delivery services are organized, staffed and delivered. This element is typically where care innovations are implemented and represents an important opportunity to improve quality of care and health outcomes of patients.
*Self-management support*	Emphasizes patient’s role in managing health. Established self-management techniques such as mutual goal setting and action planning have focused on various methods of teaching such as group classes, skill development, and various lifestyle behaviors.
*Community including organizations and resources for patients*	Involves linking and using community resources that support healthcare effort by clinicians. The use of church-based support groups, local community health programs, clinic based support groups and internet are acceptable community interventions.

The CCM called for a structural change in the way people with chronic illnesses are cared for, and the participation of nurses, social workers and patients themselves.

(b) Improving Chronic Illness Care, ICIC (Wielawski, 2011)

The idea behind ICIC is to integrate medical science with redesigned health care delivery systems so chronic patients in any setting can receive prompt diagnoses and care. Five additional themes which were incorporated into the existing CCM are described in Table 4.

**Table 4:** Additional themes included in existing *CCM*

Themes	Description
*Patient Safety (in Health System*	A system seeking to improve chronic illness care must be motivated and prepared for change throughout the organization. There is a need to identify care improvement and translate it into clear improvement goals and policies through application of effective improvement strategies, including use of incentives that comprehensive system change. Breakdowns in communication and care coordination can be prevented through agreements that facilitate communication and data-sharing as patients navigate across settings and providers.
*Cultural competency (in Delivery System Design)*	Improving health of people with chronic illness requires transformation of a system to one that is proactive instead of reactive. Roles need to be defined and tasks need to be distributed among team members. Interactions need to be planned to support evidence-based care. More complex patients may need more intensive management for a period of time to optimize clinic care and self-management. Health literacy and cultural sensitivity are two important features and providers are increasingly being called upon to respond effectively to the diverse cultural and linguistic needs of patients (Wielawski, 2011).
*Care coordination (in Health System and Clinical Information Systems)*	Effective chronic illness care is impossible without information systems that assure ready access to key data on individual patients as well as populations of patients (Wielawski, 2011; Wagner et al. 2002). An information system can identify groups of patients needing additional care as well as facilitate performance monitoring and quality improvement efforts.
*Community policies (in Community Resources and Policies)*	Mobilize community resources to meet needs of patients by advocating for policies to improve patient care.
*Case management (in Delivery System Design)*	Provide clinical case management services for complex patients and care that patients understand and that fits with their cultural background.

(c) Innovative Care for the Chronic Conditions, ICCC (WHO, 2002)

World Health Organization describes expansion of CCM to present a structure for organizing the health care for chronic conditions. The Innovative Care for the chronic conditions (ICCC) model recognizes the broader policy environment that involves patients, their families, health care organizations, and communities. Table 5 describes the eight elements of the model;

**Table 5:** Description of Elements of *ICCC*

Element	Description
*Support a paradigm shift*	A new shift will dramatically advance efforts to solve the problem of managing diverse patient demands given limited resources. Health care systems can maximize their returns from scarce and seemingly non-existent resources by shifting their services to encompass care for chronic conditions.
*Manage political environment*	Policy-making and service planning inevitably occur in a political context. Political decision-makers, health care leaders, patients, families, and community members, as well as organizations that represent them, need to be considered. It is crucial to initiate bi-directional information sharing and to build consensus and political commitment among stakeholders at each stage (Wielawski, 2011; WHO, 2002).
*Build integrated health care*	Care for chronic conditions needs integration to ensure shared information across settings and providers, and across time. Integration also includes coordinating financing across different arms of health care including prevention efforts and incorporating community resources that can leverage overall health care services. The outcome of integrated services is improved health, less waste, less inefficiency and a less frustrating experience for patients.
*Align sectoral policies for health*	The policies of all sectors need to be analyzed and aligned to maximize health outcomes. Health care can be and should be aligned with labor practices (e.g., assuring safe work contexts), agricultural regulations (e.g., overseeing pesticide use), education (e.g., teaching health promotion in schools), and broader legislative frameworks (WHO, 2002).
*Use healthcare personnel more effectively*	Health care providers, public health personnel and those who support health care organizations need new, team care models and evidence-based skills for managing chronic conditions. Advanced communication abilities, behavior change techniques, patient education, and counseling skills are necessary in helping patients with chronic problems (WHO, 2002). Health care personnel with less formal education and trained volunteers have critical roles to play.
*Center care on the patient and family*	Management of chronic conditions requires lifestyle and daily behavior change. Focusing on the patient in this way constitutes an important shift in current clinical practice. The present scenario has a patient role as a passive recipient of care, missing the opportunity to leverage what he or she can do to promote personal health. Health care for chronic conditions must be re-oriented around the patient and family.
*Support patients in their communities*	Patients and families need services and support from their communities. Communities can also fill crucial gap in health services that are not provided by organized health care.
*Emphasize prevention*	Most chronic conditions are preventable. Strategies for reducing onset and complications include early detection, increasing physical activity, reducing tobacco use, and limiting prolonged, unhealthy nutrition (Wielawski, 2011; WHO, 2002). Prevention should be a component of every health care interaction.

(d) Stanford Model (Stanford University, 2012)

The most widely used and researched self-efficacy enhancing health care intervention is Chronic Disease Self-Management Program (CDSMP) (Stanford University, 2012). The CDSMP aims to provide participants with the self-efficacy and skills required to optimally manage their chronic conditions regardless of specific diagnosis. The overall aim is to help the participants’ master six fundamental self-management tasks: solving problems, making decisions, utilizing resources, forming a patient -provider partnership, making action plans for health behavior change and self-tailoring (Stanford University, 2012).

(e) Transitional Care mode (Naylor et al., 2004)

Transitional care is defined as a set of actions designed to ensure the coordination and continuity of healthcare as patients transfer between different locations or different levels of care within the same location (Naylor et al., 2004). Representative locations include hospitals, sub-acute and post-acute nursing facilities, the patient’s home, primary and specialty care offices, and long-term care facilities. Transitional care is based on a comprehensive plan of care and the availability of healthcare practitioners who are well-trained in chronic care and have current information about the patient’s goals, preferences, and clinical status (Naylor et al., 2004). Transitional care is essential for persons with complex care needs. Transitional care model (TCM) addresses gaps in all care transitions, including hospital to home, home to hospital, physician office to home, chronic care to palliative care, and palliative care to hospice care. The TCM address patients’ chronic care needs across time including;

o Identification of patient-specific concerns related to transition process.
o Medication adherence and persistence.
o Assessing and supporting health literacy between physician visits/treatments.
o The utilization of remote patient monitoring specifically to facilitate problem solving, confidence-building, and the promotion of needed behavior changes for optimal condition management.

### 3.2 Elements of Chronic Disease Models

**Figure 3:**
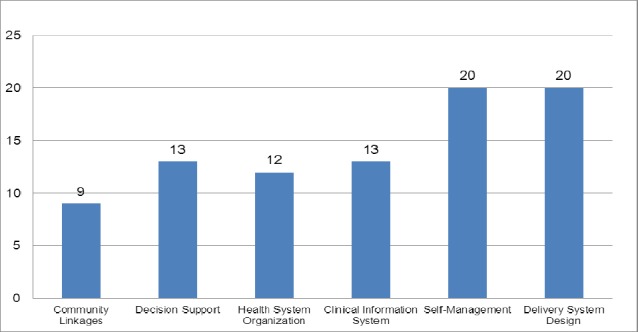
Number of studies examined elements of the various chronic disease models

The various elements of the chronic disease models that were assessed have been described in Table 6. Delivery system design and self-management support were found to be the major elements studied (n=20/23; 87%), followed by decision support and clinical information system (n=13/23; 57%), health system organization (n=12/23; 52%) and community linkages (n=9/23; 39%). Elements including center care on the patient and family (n=3/23; 13%), patient safety (n=1/23; 4%), community policies (n=1/23; 4%), built integrated health care (n=1/23; 4%) and remote patient monitoring (n=1/23; 4%) have not been well studied (Figure 3). Other elements including support paradigm shift, manage political environment, align sectoral policies for health, use healthcare personnel more effectively, support patients in their communities, emphasize prevention, identify patient specific concerns related to the transition process, and health literacy between visits and treatments have also not been well studied in the existing literature.

**Table 6:** Assessment of process variables, clinical outcomes and non clinical outcomes among various chronic disease models (Clinical outcomes included assessment of HbA1c level, blood pressure measurement, lipid measurement, adherence to treatment and self management)

Chronic Disease Model	Outcomes Changed	Process Variables N= n (Number of studies)/total studies in the review	Clinical Outcomes	Non-clinical Outcomes
**CCM**	Improved	N=9/23	N=13/23	N=12/23
	No change			N=2/23
	Not studied	N=6/23	N=2/23	N=1/23
**ICIC**	Improved	N=1/23		
	No change			
	Not studied		N=1/23	N=1/23
**Stanford**	Improved			N=1/23
	No change			
	Not studied	N=1/23	N=1/23	
**TCM**	Improved		N=1/23	
	No change			
	Not studied	N=1/23		N=1/23
**CCM + ICCC**	Improved	N=3/23	N=3/23	N=2/23
	No change		N=1/23	
	Not studied	N=2/23	N=1/23	N=3/23

**Figure 4:**
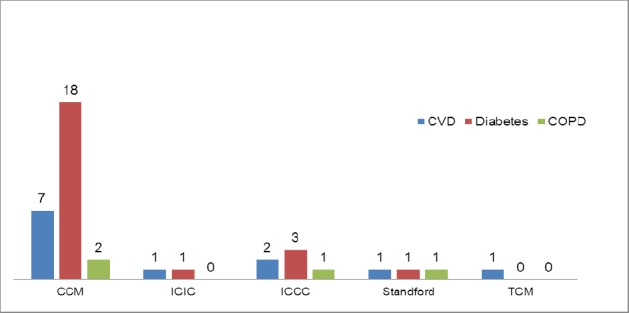
Distribution of the number of chronic disease models assessed among CVD, Diabetes and COPD

Majority of the studies utilizing chronic disease models focused on Diabetes (n=21/23; 91%), CVD (n=10/23; 43%) and COPD (n=3/23; 13%). CCM was the most studied model among all the three disease conditions: CVD (n=7/23; 30%), Diabetes (n=18/23; 78%) and COPD (n=2/3; 67%). Transitional care model was studied in only one article related to CVD (Figure 4).

**Figure 5:**
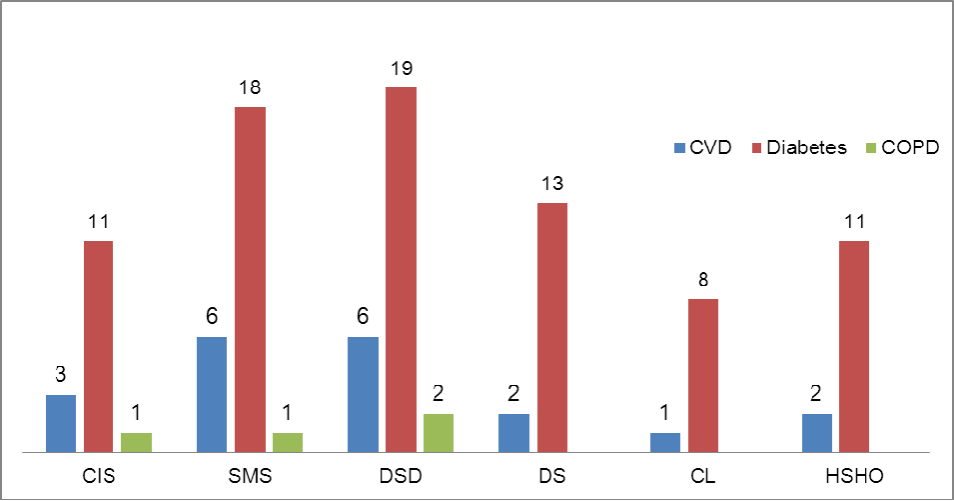
Distribution of the number of elements of the CCM studied across CVD, Diabetes and COPD

Of all the studies performed on diabetes, only one study examined the seven elements, while rest of them studied six or less elements. Among all the CVD studies, only one studied all the six elements while the remaining studied less than six elements. The specific elements of the CCM that have been studied for various chronic diseases such as CVD, Diabetes and COPD have been outlined in Figure 3. SMS (n=6) and DSD (n=6) were the most common elements of the CCM that were studied for CVD. DSD (n=19), SMS (n=18) and DS (n=13) were the most common elements of CCM studied for managing individuals with diabetes. Few studied had described the various elements of CCM to manage COPD (Figure 5).

### 3.3 Approaches Used to Implement Elements of Chronic Disease Model

***Health Care Organizations:*** Various approaches have been studied to implement element of addressing health care organizations for chronic disease management (Wagner et al. 2002; Wagner et al., 1996a; Wagner et al., 2001; Wagner et al., 1996b). Perceived team effectiveness with team champion is the backbone for the strong health care organization to provide effective management (Shortell et al., 2004; Siminerio, Zgibor & Solano, 2004). The organizational group, hierarchical and rationale culture are different cultures to which organizations value and emphasize the factors like teamwork and participation. The data on user expectations needs and satisfaction is valuable information for designing new services for any health care organization. Senior managers’ support and review work progress (Siminerio et al., 2004; Pearson et al., 2005; Piatt et al., 2011; Siminerio, Piatt & Zgibor, 2005; Piatt et al., 2010; Siminerio et al., 2006; Piatt et al., 2006). The organization needs to undergo developmental changes (Siminerio et al., 2004; Vargas et al., 2007) at various multiple levels such as involvement and participation of opinion leaders (Siminerio et al., 2004), patients (Pearson et al., 2005), and independent primary care physicians (Nutting et al., 2007) and practice nurses (Frei et al., 2010).

***Clinical Information Systems (CIS):*** Different approaches have been utilized to implement CIS for supporting chronic diseases management. CIS has shown to facilitate exchange of health information between providers and patients. The provision of flag alerts (Wellingham, Tracey, Rea & Gribben, 2003) reminders of routine events and visits (Siminerio et al., 2004; Wellingham et al., 2003) computerized clinical records like Medical Archival Retrieval System (MARS) (Siminerio et al., 2004; Tracey, & Bramley, 2003; Chin et al., 2004), and chart audits(Piatt et al.,2011; Piatt et al., 2010; Siminerio et al., 2006; Piatt et al., 2006), electronic medical records(Piatt et al.,2011; Piatt et al., 2010; Siminerio et al., 2006; Piatt et al., 2006) and internet access to contact physicians (Schmittdiel, Shortell, Rundall, Bodenheimer & Selby; 2006) are few of the mechanisms to implement CIS for chronic disease management. Patient appointment schedule can be prioritized based on their situation and can help improve communication with general practitioners (GP) (Frei et al., 2010). Patients’ diseases registry can also help to track care management of the patients (Pearson et al., 2005; Nutting et al., 2007; Chin et al., 2004). The CIS is an important and crucial way to provide tailored feedback on the performance of the organization’s chronic disease management program from patient and provider perspective (Pearson et al., 2005; Schmittdiel et al., 2006).

***Decision Support:*** Decision support is defined as evidence based guidelines consistent with scientific evidence and patient preference and should be embedded into daily practice and shared with patients to encourage participation (Wagner et al., 1996a, 1996b, 2001, 2002). Organizing problem based learning sessions for providers and patient, guideline adherence (Pearson et al., 2005; Siminerio et al., 2005; Schmittdiel et al., 2006) expert consultations (Pearson et al., 2005; Piatt et al., 2011; Siminerio et al., 2005; Piatt et al., 2010; Siminerio et al., 2006; Piatt et al., 2006; Sperl-Hillen et al., 2004), inclusion of provider education programs (WHO, 2011) and remote consultations are some of the decision support components implemented in the care of chronic disease patients (Piatt et al., 2011; Piatt et al., 2010; Siminerio et al., 2006; Piatt et al., 2006). Interactive workshops to provide evidence based information on disease management and sharing of clinical and/or management issues through use of electronic medical records are some of the other suggested approaches (Frei et al., 2010; Smith et al., 2008).

***Self-Management Support:*** It emphasizes patient’s role in managing health. Established self-management techniques such as mutual goal setting and action planning have focused on various methods of teaching such as group classes, skill development, and various lifestyle behaviors (Wagner et al., 1996a, 1996b, 2001, 2002). Personalized healthcare plan, medications, action plan, lifestyle goals and feedback for the providers to deliver tailored feedback have been studied (Pearson et al., 2005; Nutting et al., 2007; Tracey, & Bramley, 2003; Chin et al., 2004; Sperl-Hillen et al., 2004; Ciccone, 2010; Glasgow et al., 2004). Incentives have been offered to increase patients’ participation for self-management programs (Siminerio et al., 2004) Patient education, patient activation/ psychological support (Piatt et al., 2011; Piatt et al., 2010; Siminerio et al., 2006; Piatt et al., 2006; Vargas et al., 2007; Nutting et al., 2007; Schmittdiel et al., 2006; Smith et al., 2008), access to self-management resources and tools (Vargas et al., 2007; Wellingham et al., 2003; Ciccone, 2010; Schmittdiel et al., 2008; Askew, Jackson, Ware & Russell, 2010) and collaborative decision making are some of the other common components of self-management support element of CCM(Pearson et al., 2005). Individuals with chronic diseases are provided with training to improve their skills for blood glucose monitoring (Frei et al., 2010; Schmittdiel et al., 2006), adjusting insulin, and modifying diet and exercise (Schmittdiel et al., 2006), review medical charts (Schmittdiel et al., 2006) and track self- management behavior (Sperl-Hillen et al., 2004) are some of the techniques employed to improve self-management in these individuals. Only one study used Stanford model to improve self-management in chronic disease individuals (Franks, Chapman, Duberstein & Jerant, 2009).

***Delivery System Design:*** It involves understanding related to organizational design, its staffing and delivery of care services. The element involves implementation of care innovations and represents an important opportunity to improve individual quality of care and health outcomes. Delivering and exploring newer methods of health education programs in primary care settings is an important way to assess needs of people with chronic diseases (Siminerio et al., 2004). Incentives to participate in chronic disease management program (Wellingham et al., 2003) free access to services (Wellingham et al., 2003), use of computers in healthcare facilities to assess diabetes needs (Glasgow et al., 2004), holding diabetes days or diabetics clinics (Askew et al., 2010) and onsite availability of certified diabetes educator for diabetes education is another dimension of delivery systems design(Siminerio et al., 2004; Piatt et al., 2011; Siminerio et al., 2005; Piatt et al., 2010; Siminerio et al., 2006; Piatt et al., 2006; Schmittdiel et al., 2006). The delivery systems design also included planned visits, multi provider visits, and follow up of patients by case managers (Piatt et al., 2011; Piatt et al., 2010; Siminerio et al., 2006; Piatt et al., 2006; Vargas et al., 2007; Nutting et al., 2007; Schmittdiel et al., 2006), office staff, telephone and sending letters(Nutting et al., 2007). A member of the team acting as a facilitator of change and decision making is another method of delivery system design (Shortell et al., 2004; Pearson et al., 2005). Tele education intervention (Smith et al., 2008) health education classes (Schmittdiel et al., 2008), health phone (prerecorded health education audiotapes) (Schmittdiel et al., 2008), Homing in On Health (HIOH) (Franks, Chapman, Duberstein, & Jerant, 2009), home delivery variant of the peer led CDSMP to deliver health education (Franks et al., 2009) were also the means of delivery systems designs.

***Community Linkages:*** It involves linking and using community resources that support healthcare effort by clinicians. The use of church-based support groups, local community health programs, clinic based support groups and internet are acceptable community interventions (Wagner et al., 1996a, 1996b, 2001, 2002). The local community hospital and hospital system collaborations were made between university and leaders in the local community (Pearson et al., 2005; Piatt et al., 2011; Piatt et al., 2010; Siminerio et al., 2006; Piatt et al., 2006; Schmittdiel et al., 2006; Sperl-Hillen et al., 2004). Information leaflets were provided by the practice nurses to inform the patient about community resources (Frei et al., 2010).

***Cultural Competence:*** Cultural competence has been defined as a set of academic, experimental and interpersonal skills that allow individuals to increase their understanding and appreciation of cultural differences and similarities within and among groups (Wellingham et al., 2003). Only one study addressed the issue of cultural competence by holding a series of two cultural competence workshops to inform development of cultural competence section based on National Center for Cultural Competence Model (Wellingham et al., 2003).

***Centre Care on the Patient and Family:*** The studies have included patient centered elements in chronic disease management programs (Shortell et al., 2004; Ciccone, 2010; Schmittdiel et al., 2008). One of the components analyzed is to improve patient satisfaction by assessing patients’ needs and expectations by having staff promptly resolving patient complaints (Shortell et al., 2004). In another study, the care manager provided the support to the patient in implementing actions based on the prescriptions of the physicians to make the lifestyle changes (Ciccone, 2010).

***Care Coordination:*** The team based approach to disease management with care managers as a bridge between physicians, specialists and patient’s collaboration with the doctors and patients was undertaken (Ciccone, 2010). Besides this, a project management team consisting of program leader, program coordinator, and a technical resource person for day to day program operations were involved as care coordinators.

***Build Integrated Health Care (BIHC):*** It included health systems integration by involving primary care physicians, medical insurance, educators, administrators and data management systems along with the patients. An integrated system is important as it allows community physicians to have access to the many resources that private practicing physicians often lack. A central organization and coordinating structure that brings the resources of an entire system has been crucial for the success of the chronic disease programs (Siminerio et al., 2004).

***Remote Patient Monitoring:*** Remote monitoring, an element of TCM was described in one study with emphasis on CVD (Williams, Akroyd, & Burke, 2010). The clinical nurse specialist facilitated the transition of the congestive heart failure patient from hospital to home by introducing transitional care package (Williams et al., 2010). The clinical nurse specialist visited patients regularly in the wards and provided information to them about their health condition to help them better prepare for discharge. This approach increased self-efficacy of the patients thereby developing their confidence to make decisions about their health (Williams et al., 2010).

### 3.4 Outcomes Assessed

The outcomes were categorized as process, clinical and non-clinical outcomes. More than half of the studies assessed process variables (57%, n=13/23). About 78% (n=18/23) of them studied clinical outcomes and 74% (n=17/23) studied non clinical outcomes. The most common process variables included completion of diabetes tests based on evidence based guidelines (Piatt et al., 2011; Siminerio et al., 2005; Nutting et al., 2007; Frei et al., 2010; Chin et al., 2004; Smith et al., 2008; Ciccone, 2010; Glasgow et al., 2004), percentage of patients receiving at least one eye examination per year (Piatt et al., 2011; Siminerio et al., 2005; Frei et al., 2010; Chin et al., 2004; Ciccone, 2010; Glasgow et al., 2004), percentage of patients receiving at least one foot examination per year (Piatt et al., 2011; Siminerio et al., Frei et al., 2010; 2005; Ciccone, 2010), percentage of patients receiving at least one nephropathy screening per year (Siminerio et al., 2005; Nutting et al., 2007; Frei et al., 2010; Ciccone, 2010) percentage of patients receiving at least one neurological testing (Siminerio et al., 2005; Nutting et al., 2007; Ciccone, 2010) and routine lipid test rates (Siminerio et al., 2005; Nutting et al., 2007; Frei et al., 2010; Chin et al., 2004; Smith et al., 2008; Ciccone, 2010; Glasgow et al., 2004).

**Figure 6:**
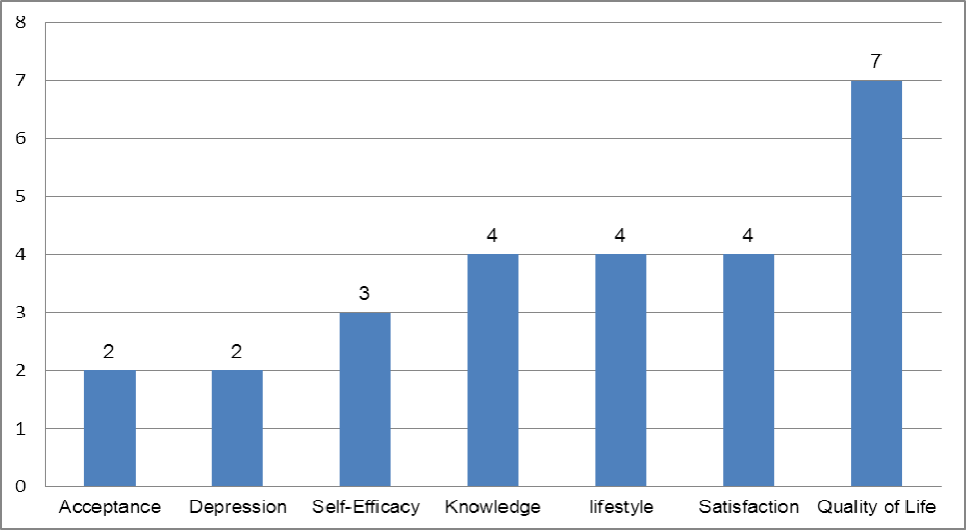
Most common non clinical outcomes assessed by the number of studies

The most common clinical outcomes evaluated included HbA1C (56%, n=13/23), lipid measurements (48%, n=11/23), blood pressure measurements (43%, n=10/23), adherence to treatment care (26%, n=6/23) and self-management (13%, n=3/23). The most common non–clinical outcomes evaluated included quality of life, patient and provider satisfaction and modification in lifestyle behaviors (including diet and physical activity) as shown in Figure 6.

Majority of the studies showed improvement in process variables (39%, 9/23) and clinical outcomes (56%, n=13/23) (Table 6).

## 4. Discussion

Although implementation of the chronic disease models has been associated with important improvements in measures of processes of chronic care, it has not always been associated with improvement in intermediate or long term outcomes. The most challenging CCM element to implement in primary care has been clinical information systems (Smith et al., 2008). Missed opportunities in medication management could explain the paradox of improved system performance without improved patient outcomes. This can be resolved through enhanced information and decision support to the primary care team (Smith et al., 2008). Numerous well designed attempts to improve delivery of preventive services have been made, but a few have proven to be broadly applicable or successful. Major barriers to success are numerous competing demands placed upon primary care offices and the very limited amount of time available (Nutting, Rost, Smith, Werner & Elliot, 2000). To increase chances of adoption and success, an intervention should be brief, fit into the flow of patient visits, not increase the time demands on physician time, and inform the patient-provider interaction.

There was a reluctance of all parties being a part of development process and key policy decisions. An underlying need for the governance group to be functional is to delegate financial responsibility and in exchange carries outcome accountability (Wellingham et al., 2003). Achieving health outcomes is seen as an output of negotiated decisions between the patients, in context of their normal environment and health advisory team. For patients with chronic disease to have better health outcomes they need to feel understood, respected and empowered by the general practice team (Wellingham et al., 2003).

Chronic illness care can be improved, if delivery system adopts a primary health care orientation emphasizing comprehensives of care and the overall health of the patient (Schmittdiel et al., 2006). The traditional health care system is designed to provide a symptom driven response to acute illnesses, it is poorly configured to meet the needs of those with chronic illnesses (Siminerio et al., 2005).

Evidence suggests that the application of CCM principles to health care systems lead to better outcomes for patients with chronic illnesses (Schmittdiel et al., 2006). The principles include (i) first contact (primary care physicians should be patient’s first contact), (ii) continuity includes relationship between the primary care physician and patient should be long term and consistent over time, (iii) comprehensiveness should provide a wide range of preventive and acute care services to meet a large proportion of patient medical records, (v) coordination involves primary care systems to coordinate care across physicians, ideally using electronic information systems and (v) accountability includes primary care physicians to be responsible for patient’s overall health and medical outcomes (Schmittdiel et al., 2008). Results also confirm findings from previous studies suggesting a gap between the reference standards of chronic illness care and the level of chronic care management processes provided by physician organizations (Schmittdiel et al., 2008). This pilot study demonstrates the feasibility and usefulness of implementing elements of the CCM into a rural practice site. The primary care providers in a rural practice identified lack of education services, psychological and psychosocial factors as major barriers to care (Schmittdiel et al., 2006). It is important to explore these elements to obtain information for the development of programs specific to the locality.

Effective chronic disease programs ensure access to providers for decision support facilitated though evidence based guidelines and to patients for self-management education and team based care. Prior studies have shown that patients are not participating in preventive health care services such as education and team care is rarely available or employed in primary care. Facilitating effective chronic illness management often requires planned care and changes in delivery system design(Schmittdiel et al., 2006). Planned care within a redesigned healthcare system can improve care delivery for people with chronic diseases.

One of the major problems addressed by the CCM is the fact that current care of chronically ills is often reactive and triggered by actual problems instead of being proactive, structured and planned (Frei et al., 2010). To perform care according to the CCM, a team approach involving the practice nurse is required. This represents a further challenge since practice nurses are currently only marginally involved in the care for patients. To reduce readmissions and length of stay in hospital, nurses need to take an increased lead within the multidisciplinary team in helping to safely discharge patients from the hospital (Williams et al., 2010). Other challenges include needing more time and resources, difficulty developing computerized patient registries, team and staff turnover, and occasional need for more support by senior management (Chin et al., 2004). Currently few efforts exist to implement quality of care in diabetes despite studies that demonstrate their proven effectiveness (Piatt et al., 2006).

Community partnerships, population based sample of participants, flexible, patient centered approach and primary care practice redesign suggests that CCM for improving diabetes care is feasible and effective (Piatt et al., 2006). Areas that were most challenging in the implementation of CCM were concerned time and the related burden of data collection and report generation. The only one year randomized controlled trial of the CDSMP involved the internet-delivered variant of the program (Franks et al., 2009). The study also found a significant short-term increase effect on disease management self-efficacy, but again the effect was no longer significant by one year. Taken together these findings suggest the self-efficacy enhancing effects of the CDSMP are relatively short lasting (Franks et al., 2009). Greater implementation of the CCM enables patients to engage in more self-management behaviors (Schmittdiel et al., 2008). The CCM has explicitly been suggested as template for the care for chronically ills in Germany. The resources regarding medical professionals as nurse practitioners are completely different in Switzerland compared to the U.S. but these professionals play an important role in the CCM. So far, no experiences are available with implementation of the CCM in the Swiss healthcare system (Frei et al., 2010).

There are several limitations of our study. One of the limitations of this systematic review could possibly be inclusion of only those studies that specifically mention a particular chronic disease model. There are individual elements of these chronic disease models that have been widely studied in literature but have not referred to and hence might have been excluded from the final analysis. This might have limited the number of studies that might be otherwise relevant. In some of the studies, the elements of the chronic disease model were difficult to classify and hence were subject to authors’ judgment. This could have possibly resulted in some error in classifying these elements.

Future studies are needed to test chronic disease models in settings where more racially and ethnically representative patients receive chronic care. Future program development should also include information on other barriers including transportation issues, finances and lack of services. It is unclear if and to what extent the results generated in the U.S settings are transferrable to Europe or other Asian countries. Further, it becomes critical to define the different elements of the chronic disease model and their methods of implementation in a much more definitive manner so that they can be adapted at multiple levels of care across different settings to assess their generalizability.
